# Association Between Primary Hyperparathyroidism and Secondary Diabetes Mellitus: Findings From a Scoping Review

**DOI:** 10.7759/cureus.40743

**Published:** 2023-06-21

**Authors:** Maxim J Barnett

**Affiliations:** 1 Internal Medicine, Einstein Medical Center Philadelphia, Philadelphia, USA

**Keywords:** primary hyperparathyroidism, parathyroidectomy, hyperparathyroidism, insulin resistance, diabetes mellitus

## Abstract

An ill-defined association exists between diabetes mellitus (insulin resistance) and primary hyperparathyroidism. This article explores this phenomenon while providing an explanation for such a relationship and reviewing the evidence regarding the response to insulin sensitivity following a parathyroidectomy. Primary hyperparathyroidism may increase the risk of developing insulin resistance; peculiarly, this is not present in all patients. It is likely that both intracellular hypercalcemia and hypophosphatemia alter the insulin receptor expression and response; the contribution of parathyroid hormone is less clear. Following parathyroidectomy, patients may demonstrate improvement in their insulin sensitivity, while others have no response or a detrimental effect. A varied phenotype exists among patients, and furthermore, it is unclear why certain patients demonstrate improvement in insulin sensitivity following a parathyroidectomy, whereas others fail to do so. While this review provides a broad overview of the general endocrine community, it is imperative to note that clinical applicability is limited until further studies address these remaining uncertainties. Due to the lack of understanding regarding this endocrinological enigma, the presence of insulin resistance, at this present time, should not be a criterion for a parathyroidectomy.

## Introduction and background

Primary hyperparathyroidism, an endocrinological disorder characterized by high calcium and inappropriately elevated parathyroid hormone (PTH), is associated with a heightened cardiovascular risk profile. The associations that are described in the literature include an increase in body mass index (BMI), elevated low-density lipoprotein cholesterol (LDL-C), decreased high-density lipoprotein cholesterol (HDL-C), elevated high-sensitivity C-reactive protein (a marker of vascular inflammation), and impairment in insulin sensitivity (with ensuant hyperglycemia) [1]. This latter phenomenon, which is referred to as insulin resistance, is an area of growing interest within the current medical literature; it is notable that around 8% of patients with primary hyperparathyroidism may demonstrate diabetes mellitus, which is a three-to-four-fold greater increase than that of the general reference population. Conversely, however, no more than 1% of patients with diabetes mellitus may demonstrate overt primary hyperparathyroidism, which suggests that states of insulin resistance may occur as a secondary manifestation of hyperparathyroidism [1]. A further area of uncertainty in the field of endocrinology is whether or not the management of primary hyperparathyroidism (with a parathyroidectomy) leads to either improvement or a resolution of the state of insulin resistance. It is the purpose of this article therefore, to review the medical literature to either describe or refute such a relationship between primary hyperparathyroidism and diabetes mellitus (or a state of insulin resistance), moreover, to investigate the proposed pathophysiology which suggests such a bond between these two endocrinological disorders, and to ultimately provide an overview regarding the current literature in order to determine whether or not a parathyroidectomy (for management of primary hyperparathyroidism) leads to an improvement in ones’ glycemic status.

The linkage between primary hyperparathyroidism and diabetes mellitus remains ill-defined, likely due to the limited awareness of this apparent connection in the medical community. The result of this knowledge gap is that today, much of the current literature available is posed only as ‘speculative’ reports. Due to the limited available data, as well as the significant uncertainties surrounding this topic, this article is provided as a scoping review that will analyze all available published, as well as unpublished, data, with the overall intent to collate findings conferring (or refuting) the aforementioned endocrinological relationship. An additional aim is to provide a plausible pathophysiological hypothesis. A scoping review is chosen as the method of review as it allows areas of uncertainty within the medical literature to be highlighted, and additionally, pinpoints areas that require future studies to address at a later date. Given that a narrative review provides an overview of the data, rather than an in-depth study of the subject matter, neither a risk of bias assessment nor a quality appraisal for each article is performed. It is a well-established fact that the highest-regarded bodies of evidence capable of influencing clinical decision-making are randomized controlled trials, systematic reviews, and meta-analyses. Given that a theoretical endocrine enigma is infrequently addressed, a scoping review is most appropriate to cover such less-regarded bodies of evidence. For this reason, all forms of published articles are encompassed within the article.

The primary objective is to investigate the evidence denoting a temporal relationship between both primary hyperparathyroidism and diabetes mellitus (or states of insulin resistance), with the outcome of broadening the awareness of this phenomenon throughout both the endocrine and the non-endocrine communities. The secondary objectives are to review plausible pathophysiological theories for such a relationship, and, in addition, to explore whether a parathyroidectomy may improve states of insulin resistance.

## Review

Epidemiology

Kumar and Singh [2] note that around 40% of patients with primary hyperparathyroidism may demonstrate impaired glucose tolerance (as opposed to overt diabetes mellitus), findings which are consistent with the work by Irvin et al. [3], who note that up to 54% of their patients with primary hyperparathyroidism demonstrate diabetes mellitus when analyzed pre-operatively. Taylor and Khaleeli [1] note a prevalence of 7.8% of diabetes mellitus in patients with primary hyperparathyroidism, which is similar to the findings by Cardenas et al. [4] of 15%; Ljunghall et al. [5] of 8.2%; and Valdemarsson et al. [6] of 9.4%. Cardenas et al. [4] note that the greater incidence of diabetes mellitus was only significant in the higher age bracket, with ages ranging from 64 to 75 years; however, following standardized prevalence ratios, there was only a significant difference in prevalence among men. The authors note that only age and BMI are significant risk factors for developing diabetes mellitus in patients with primary hyperparathyroidism.

Kumar and Singh [2] note that primary hyperparathyroidism presents prior to diabetes mellitus in 20% of the cases, with diabetes mellitus occurring prior to primary hyperparathyroidism in 40%, and both presenting together (or within one year of each other) in 40% of cases. Walsh et al. [7] pose a constellation of eight patients with coexistent primary hyperparathyroidism and diabetes mellitus, noting that for most patients, diabetes mellitus was diagnosed first, followed by primary hyperparathyroidism. The authors note that the diabetes mellitus was likely ‘masking’ the primary hyperparathyroidism (and therefore, it cannot be assumed that the diabetes mellitus was the first to occur internally); primary hyperparathyroidism was only considered later, after the patients' fatigue and weight loss continued, despite optimal medical management with oral anti-hyperglycemic agents. This finding is reproduced by the work of Kumar and Singh [2], who propose that it is likely that diabetes mellitus is identified first (even though it is not necessarily present first) in the literature. It is also possible that the coexistence of both primary hyperparathyroidism and diabetes mellitus is present at an even greater frequency than anticipated but is not necessarily investigated due to the commonality of symptoms among both conditions (these symptoms can include thirst, polyuria, fatigue, and weight loss). Due to the similar presentation of both primary hyperparathyroidism and diabetes mellitus, Kumar and Singh [2] recommend ruling out primary hyperparathyroidism in patients with diabetes mellitus who complain of some or all of these vague symptoms. Halver [8] notes that PTH increases renal tubular glucose reabsorption and notes that if urine testing is relied upon for screening for diabetes, mild cases may be overlooked in such a cohort. Ljunghall et al. [5] advise caution however, noting that this may simply be a demonstration of Berkson’s Bias (a type of selection bias whereby the general population is not represented in the sample being analyzed), as patients with diabetes mellitus are likely to undergo greater routine biochemical screening than the healthy population.

Etiology and hypotheses

Prager et al. [9] note that patients with primary hyperparathyroidism have both a diminished glucose-lowering effect of intravenous insulin compared with controls (p < 0.01) and insulin binding (p < 0.01), which the authors conclude is likely due to the downregulation of insulin receptors. Kumar et al. [10] demonstrate that patients with primary hyperparathyroidism attain higher plasma glucose following an infusion compared to controls (p < 0.04), with 42% of patients with primary hyperparathyroidism having impaired glucose tolerance and lower insulin sensitivity (60.3%; p < 0.001). The authors furthermore demonstrate a reduction in beta cell function in patients with both primary hyperparathyroidism and impaired glucose tolerance compared to patients with primary hyperparathyroidism and normal glucose tolerance (p < 0.05). Yasuda et al. [11] assess the response following a 100-g oral glucose load in patients with primary hyperparathyroidism, noting a linear relationship between serum calcium and calculated insulin area (r = 0.81, p < 0.001).

**Figure 1:**
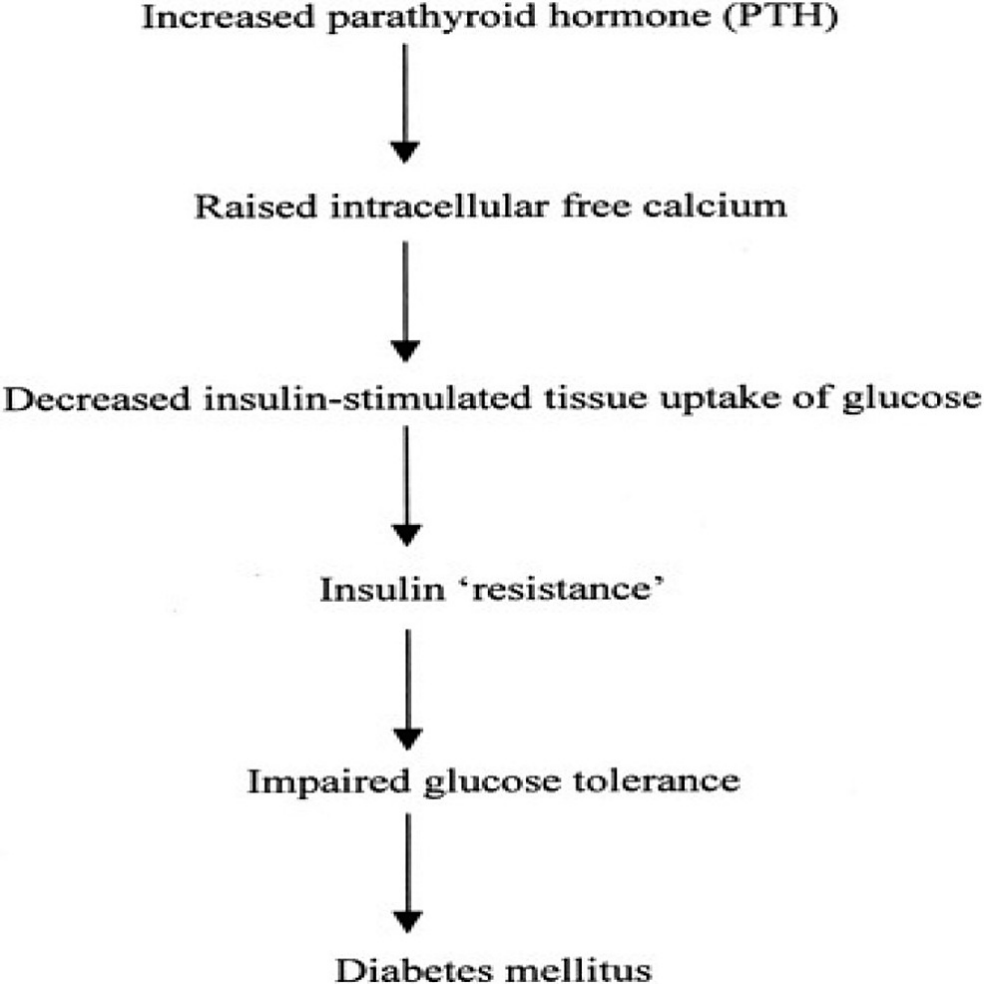
Pathophysiology of Hyperparathyroidism and Related Diabetes Mellitus From Taylor and Khaleeli [1].

As noted previously, patients with primary hyperparathyroidism are likely to demonstrate elements of the metabolic syndrome; as demonstrated by Bolland et al. [12], patients with primary hyperparathyroidism are on average 3.34 kg heavier than controls (p < 0.000001). Patients with primary hyperparathyroidism are at a heightened risk for both cardiovascular mortality and morbidity, which could likely be explained by the greater incidence of diabetes mellitus. Hagström et al. [13] concur with such findings, noting that patients with primary hyperparathyroidism have greater serum glucose, LDL-C, very-low-density-lipoprotein cholesterol, triglycerides and BMI, compared to controls (p < 0.0001-0.035), as well as diminished HDL-C (p = 0.013). Hagström et al. [13] further demonstrate that it is the calcium (both total and ionized) which negatively correlates with BMI, LDL-C, and triglycerides (p = -0.43-0.050; p = -0.5; p = 0.007); however, the authors fail to identify a significant correlation between PTH and the investigated variables, suggesting that the variable effects are solely due to calcium. Luboshitzky et al. [14] pose that it is the calcium that significantly predicts the risk of metabolic syndrome (odds ratio: 1.875, 95% CI: 1.259-2.793, p = 0.002) and that of insulin resistance (odds ratio: 2.043, 95% CI: 1.365-3.057, p = 0.002). A leading hypothesis, suggested by Kumar and Singh [2], is that increased intracellular calcium interferes with tyrosine kinase activity (of the insulin receptor) (Figure 1) [1]. Jang et al. [15] describe the relationship of calcium to insulin secretion, noting a reduction in plasma insulin following the administration of calcium channel blockers in patients with features of metabolic syndrome. An additional theory is that of recurrent pancreatitis, a known consequence of primary hyperparathyroidism, for which long-term damage could explain the incidence of diabetes mellitus; however, this requires a prolonged course, which is unlikely to be missed clinically.

In healthy subjects (without primary hyperparathyroidism), Becerra-Tomás et al. [16] demonstrate that each 1 mg/dL increase of albumin-adjusted calcium leads to a hazard ratio of diabetes incidence of 2.87 (95% CI: 1.18-1.96; p < 0.02). Wu et al. [17] demonstrate that calcium has a significant correlation between homeostatic model assessment for insulin resistance (HOMA-IR) and peripheral insulin resistance (r = 0.195, p < 0.0001; r = -0.134, p < 0.0001). Sun et al. [18] claim a positive correlation between serum glucose and calcium in men and women (r = 0.31; r = 0.22, p < 0.001); interestingly, only in women did Sun et al. [18] identify a positive correlation between serum insulin and calcium as well as an inverse association between serum calcium and beta cell function. The authors expand upon this latter finding, noting the existence of an inverse correlation between beta cell function and calcium, only in pre-menopausal (but not in post-menopausal) women (r = -0.18, p < 0.001) [18]. A further point of interest is the work posed by Reis et al. [19], noting that the association of primary hyperparathyroidism and diabetes mellitus is not apparent in black participants, reasons for which are unclear and not further addressed within the medical literature. Considering primary hyperparathyroidism is more common among black (post-menopausal) patients, this is an area that demands further research.

Al-Jehani et al. [20] note that in primary hyperparathyroidism, HOMA-IR is positively correlated with both calcium and BMI (p < 0.001), but negatively associated with phosphate. Haap et al. [21] further demonstrate (in healthy subjects without a diagnosis of diabetes mellitus) a negative correlation between phosphate and post-prandial two-hour glucose (r = -0.13, p < 0.0001), in addition to a positive correlation between insulin sensitivity and phosphate (r = 0.10, p = 0.0006); a plausible explanation for these significant associations is that low phosphate leads to depleted intracellular adenosine triphosphate, which may alter intracellular energy metabolism. Akter et al. [22] further note that low phosphate leads to insulin resistance and impaired fasting glucose; the authors note an inverse correlation between serum phosphate and calcium-phosphate products among HOMA-IR (p < 0.01). Similarly, Lorenzo et al. [23] demonstrate that calcium (odds ratio: 1.26; 95% CI: 1.04-1.53) and calcium-phosphate products (odds ratio: 1.29, 95% CI: 1.04-1.59) are associated with incident diabetes.

Certain authors, however, argue that the incidence of insulin resistance in primary hyperparathyroidism is due to the elevation of PTH, and not, as the researchers above have stated, due to the elevation of calcium. Chiu et al. [24] demonstrate in healthy subjects that plasma intact PTH is inversely correlated with the insulin sensitivity index (when using the hyperglycemic clamp (p = 0.019)), in addition to a positive correlation between plasma intact PTH and first-phase insulin release (but not the second); the authors conclude that for 1 pg/mL increase in plasma intact PTH, there is a concurrent decrease in insulin sensitivity of 0.247 mmol/L/m^2^/min/pmol/L. Røislien et al. [25] note that a one-unit standard deviation increase in PTH leads to an increase of 0.36 log odds of metabolic syndrome. Antonopoulou et al. [26] further note a positive correlation between homeostasis model assessment B (HOMA-B) and PTH (r = 0.53, p = 0.008), in addition to a correlation between PTH and waist-to-hip ratio (r = 0.44, p = 0.03), and an inverse correlation between PTH and fasting plasma glucose/fasting insulin ratio (r = -0.40, p = 0.056). After linear regression, the authors continue to show a significant linear relation between HOMA-B and PTH (p = 0.006). Karras et al. [27] assess patients with normocalcemic primary hyperparathyroidism, noting continued unfavorable glycemic profiles, from which the authors conclude is a result of elevated PTH and not elevated calcium (as normocalcemic primary hyperparathyroidism requires serum calcium to be within the reference range). Cardenas et al. [4] note that elevated PTH may lead to hepatic glucose production (both in vitro and in animal studies). Valdemarsson et al. [28] additionally demonstrate that primary hyperparathyroidism is associated with elevated plasma levels of islet amyloid polypeptide (a misfolded protein found in the pancreas of patients with type 2 diabetes mellitus).

Not all authors demonstrate a significant association between primary hyperparathyroidism and diabetes mellitus incidence; this is possibly explained by Luboshitzky et al. [14], who note that this may depend upon the ‘severity’ of hyperparathyroidism. As noted by Luboshitzky et al. [14], those with severe primary hyperparathyroidism have the greatest likelihood of cardiovascular risks, metabolic syndrome, and insulin resistance (odds ratio: 3.7, 95% CI: 1.64-8.29, p = 0.002). Moreover, there is significant disagreement among authors as to whether or not a parathyroidectomy improves the glycemic profile (and furthermore, whether or not the presence of insulin resistance should be a criterion for a parathyroidectomy in a patient with primary hyperparathyroidism). Currently, as demonstrated by Bilezikian [29], the recommendations for surgery in primary hyperparathyroidism include age below 50 years; serum calcium more than 1 mg/dL above the upper reference range; renal manifestations (creatinine clearance below 60 mL/minute, hypercalciuria of more than 400 mg/day, nephrolithiasis or nephrocalcinosis); and skeletal manifestations (vertebral fracture on imaging or reduced bone mineral density with a T-score of below -2.5). As demonstrated, insulin resistance does not appear as an indication for a parathyroidectomy.

Treatment

Certain authors, such as Kumar and Singh [2] and Walsh et al. [7], present case reports whereby patients with diabetes mellitus effectively either reduce their requirements or discontinue their insulin altogether following parathyroidectomy. Khaleeli et al. [30] demonstrate that following a parathyroidectomy, fasting and two-hour post-prandial glucose levels are significantly reduced (p < 0.05, p < 0.01) in addition to a 50% reduction in diabetes mellitus frequency, and a 33% reduction in the frequency of impaired glucose tolerance/impaired fasting glucose (along with a 35% increased frequency of normal glucose tolerance). Antonopoulou et al. [31] note that following a parathyroidectomy, a positive correlation remains between PTH and HOMA-2 in regard to beta cell function (pre-operatively r = 0.74, p = 0.02; post-operatively r = 0.55, p = 0.04). Antonopoulou et al. [26] note that HOMA-B and PTH remain significant following such surgery (r = 0.76, p = 0.002). Duran et al. [32] further note that at two months post-parathyroidectomy, there is a reduction in insulin resistance (HOMA-IR) (p = 0.003) and serum insulin (p = 0.003), in addition to noting a positive correlation between pre-operative calcium and both pre-operative insulin (r = 0.480, p = 0.028) and HOMA-IR (r = 0.478, p = 0.028).

Conversely, Ljunghall et al. [5] note in their study that, post-operatively, nearly one-third of patients demonstrate a deterioration of glucose control with pathological values. Lundstam et al. [33], as part of the Scandinavian Investigation of Primary Hyperparathyroidism (SIPH) trial, note no difference in glucose metabolism after a parathyroidectomy at five years following randomization. Richards and Thompson [34] argue that the presence of diabetes mellitus should be an indication for a parathyroidectomy, as 37% of their patients had improvements with glucose control (and another 40% remained stable); it should be noted however, that in 23% of the cases, the glucose control paradoxically worsened following surgery. The explanation as to why a paradoxical worsening occurred is unclear, and this is therefore an area that also demands to be addressed further. Bannon et al. [35] review 36 patients who are insulin-dependent and undergo a parathyroidectomy, noting no change in insulin requirement pre-and post-operatively; the result from this finding leads the authors to strongly advise against the presence of diabetes mellitus (or insulin resistance) as a criterion for a parathyroidectomy. Rudman et al. [36] fail to identify a change in insulin resistance at five and six weeks following a parathyroidectomy; however, in the subgroup analysis, those with a higher pre-operative HOMA-IR (more than 1.2 compared to less than 1.0) demonstrated an improvement. From this finding, the authors conclude that a parathyroidectomy may be more beneficial in those for whom insulin resistance is highest pre-operatively (such as the cohort depicted by Luboshitzky et al. [14]); this 'stratification' is not performed in most studies and may help to explain why certain patients' insulin resistance improves following parathyroidectomy, whereby others fail to do so.

Cakir et al. [37] analyze patients with normocalcemic primary hyperparathyroidism, and from their analysis, recommend against a parathyroidectomy, as the findings from their study do not demonstrate an improvement in either insulin resistance or glucose intolerance. Similarly, Hagström et al. [13] note that a parathyroidectomy does not influence fasting glucose or HbA1c in normocalcemic primary hyperparathyroidism. The work of Cakir et al. [37] differs from Karras et al. [38], in that there is a significant improvement in fasting (p = 0.021) and two-hour post-prandial glucose (p = 0.041) concentrations following a parathyroidectomy in patients with normocalcemic primary hyperparathyroidism.

## Conclusions

It remains unclear as to why certain patients with primary hyperparathyroidism develop diabetes mellitus (or overt insulin resistance), whereas others do not. The pathophysiological explanation is not fully elucidated; however, a leading theory is that of increased intracellular calcium leads to the interruption of post-insulin signaling. While insulin may be increased during primary hyperparathyroidism, it is noted that patients are likely to be paradoxically resistant. A further area of uncertainty addressed in the article is whether a parathyroidectomy leads to an improvement in ones’ glycemic profile; it is also unclear as to why certain patients may respond to this surgery, while others do not.

It is important to consider the logical fallacies with the aforementioned studies; not all take into account body weight, family history of diabetes mellitus, the severity of primary hyperparathyroidism, duration of primary hyperparathyroidism, normocalcemic versus hypercalcemic primary hyperparathyroidism, or the cause of the primary hyperparathyroidism, all of which are factors that may explain the differing results above. Moreover, certain studies solely assess ‘glucose levels’, whereas others assess insulin secretion, sensitivity, and resistance, which could again explain the differing results. One further consideration is the diagnostic criteria used for diabetes mellitus, which is different among the global studies over the past decades. Currently, there is not enough data to definitively provide a recommendation, and therefore, the presence of insulin resistance should not be used as a criterion for patients to undergo a parathyroidectomy until future studies adequately address the uncertainties mentioned above.
